# Supplementary material for: Sediment controls dynamic behavior of a Cordilleran Ice Stream at the Last Glacial Maximum
Source: Nat Commun. 2020 Apr 14;11:1826. doi: 10.1038/s41467-020-15579-0 (PMC7156478; doi:10.1038/s41467-020-15579-0)
Supplement: Supplementary file 1 — Supplementary Information [file 41467_2020_15579_MOESM1_ESM.pdf]

Supplementary Information for:

Sediment controls dynamic behavior of a Cordilleran Ice Stream at the Last Glacial Maximum

by Cowan *et al.*

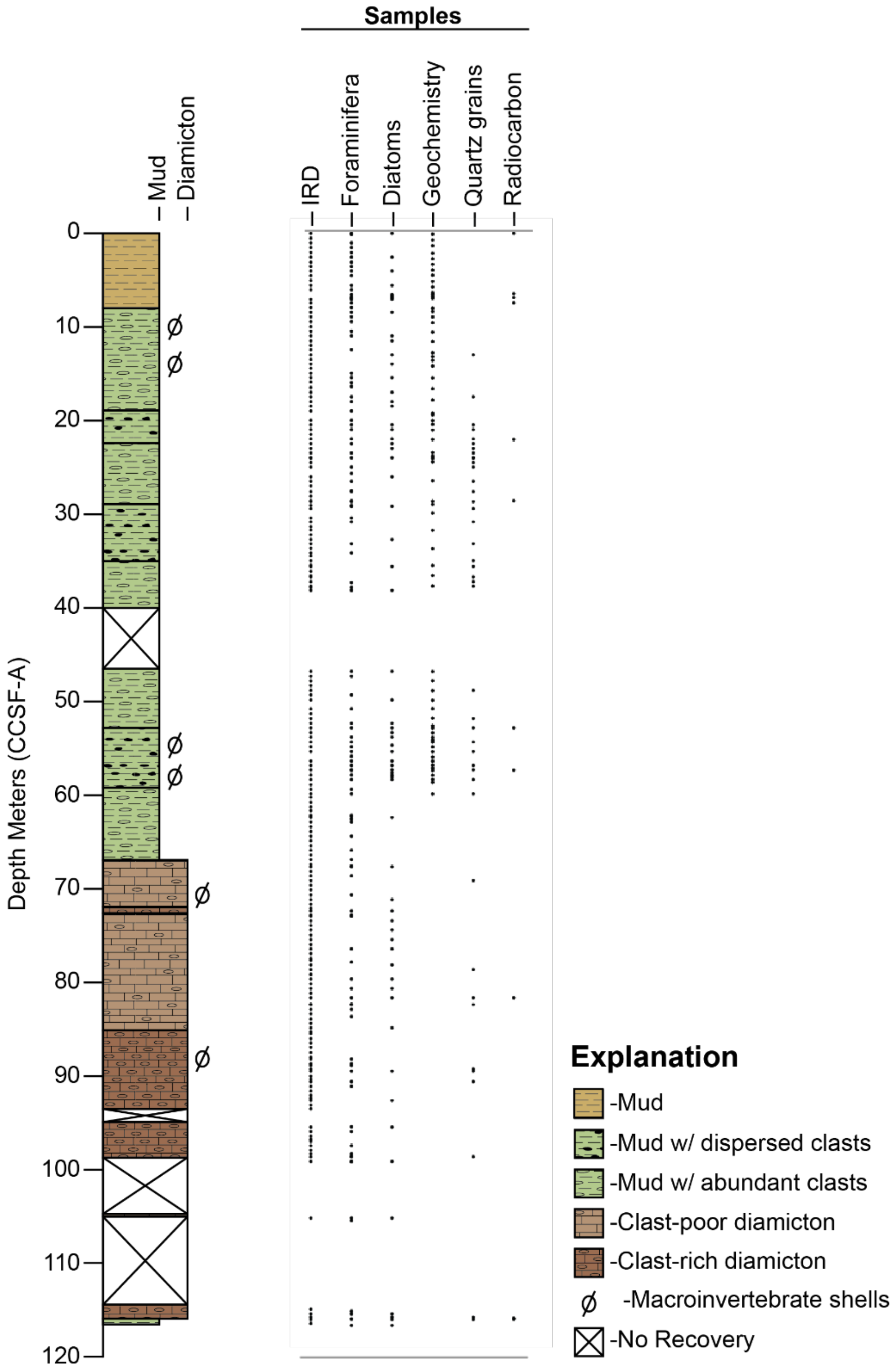

Supplementary Figure 1. Lithology log of the composite section studied from 0 to 116.5 m (CCSF-A) at Site U1421. Samples analyzed for analyses are plotted against depth (midpoint CCSF-A).

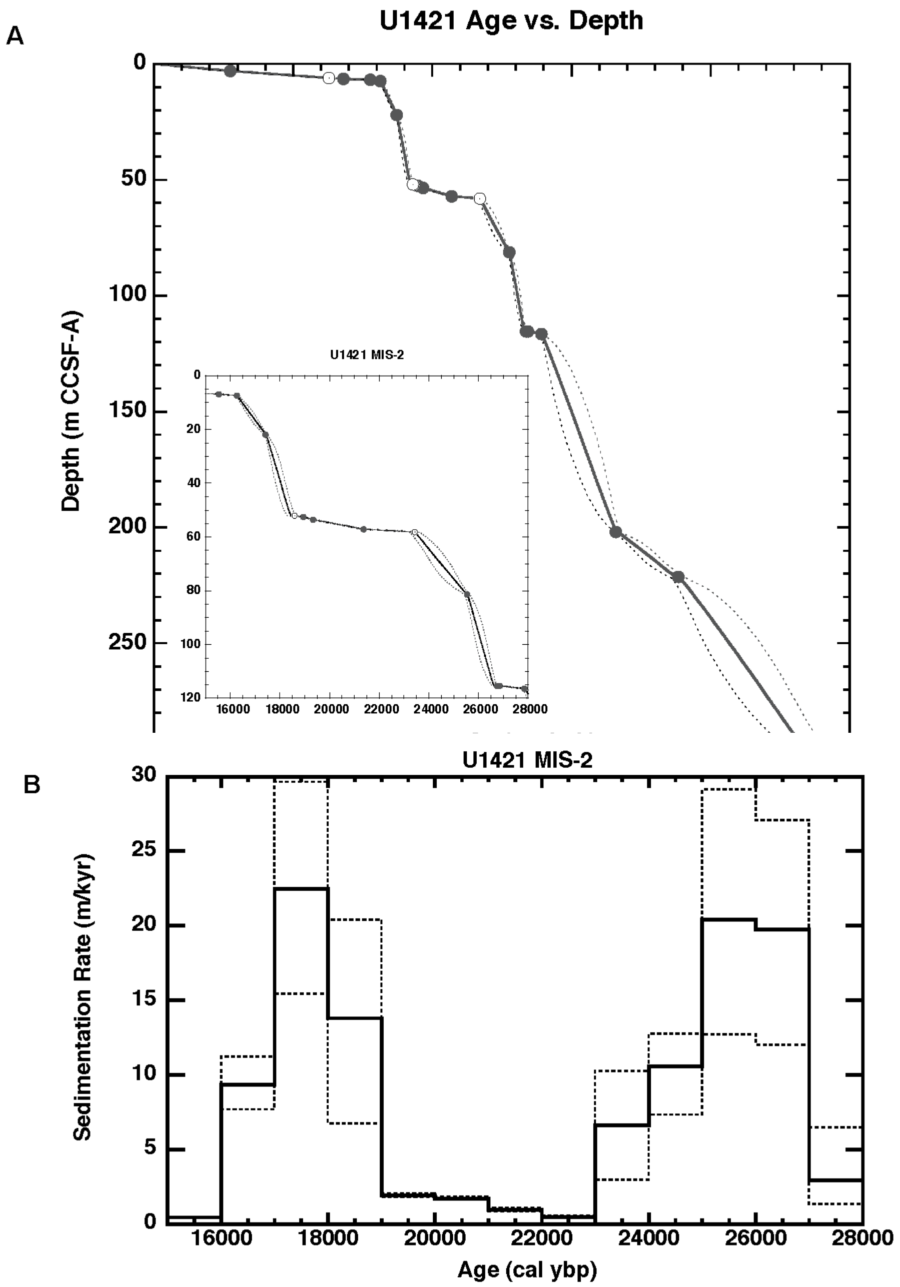

Supplementary Figure 2: **a** Bayesian age-depth model[65] for U1421 showing mean (solid line) as well as +/- 1-sigma uncertainty (dashed lines). The age model is constrained by foraminiferal radiocarbon dates (solid circles; all of which are planktic save the uppermost date which is benthic), calibrated via Marine13 assuming a DR equivalent to modern values of 470 +/- 80 years, as well as via three lithologic boundaries interpreted as constraints on transitions in sedimentation rate (open circles). The inset panel highlights the period of Marine Isotope Stage 2 (MIS-2) including the Last Glacial Maximum, which is the focus of this manuscript. **b** Sedimentation rates through MIS-2 calculated from the 10,000 Monte-Carlo simulations of the BChron Bayesian age model including mean (solid line) as well as +/- 1-sigma uncertainty (dashed lines), binned to a constant resolution of 1000 years to remove artifacts superimposed by the variable density of chronological constraints.

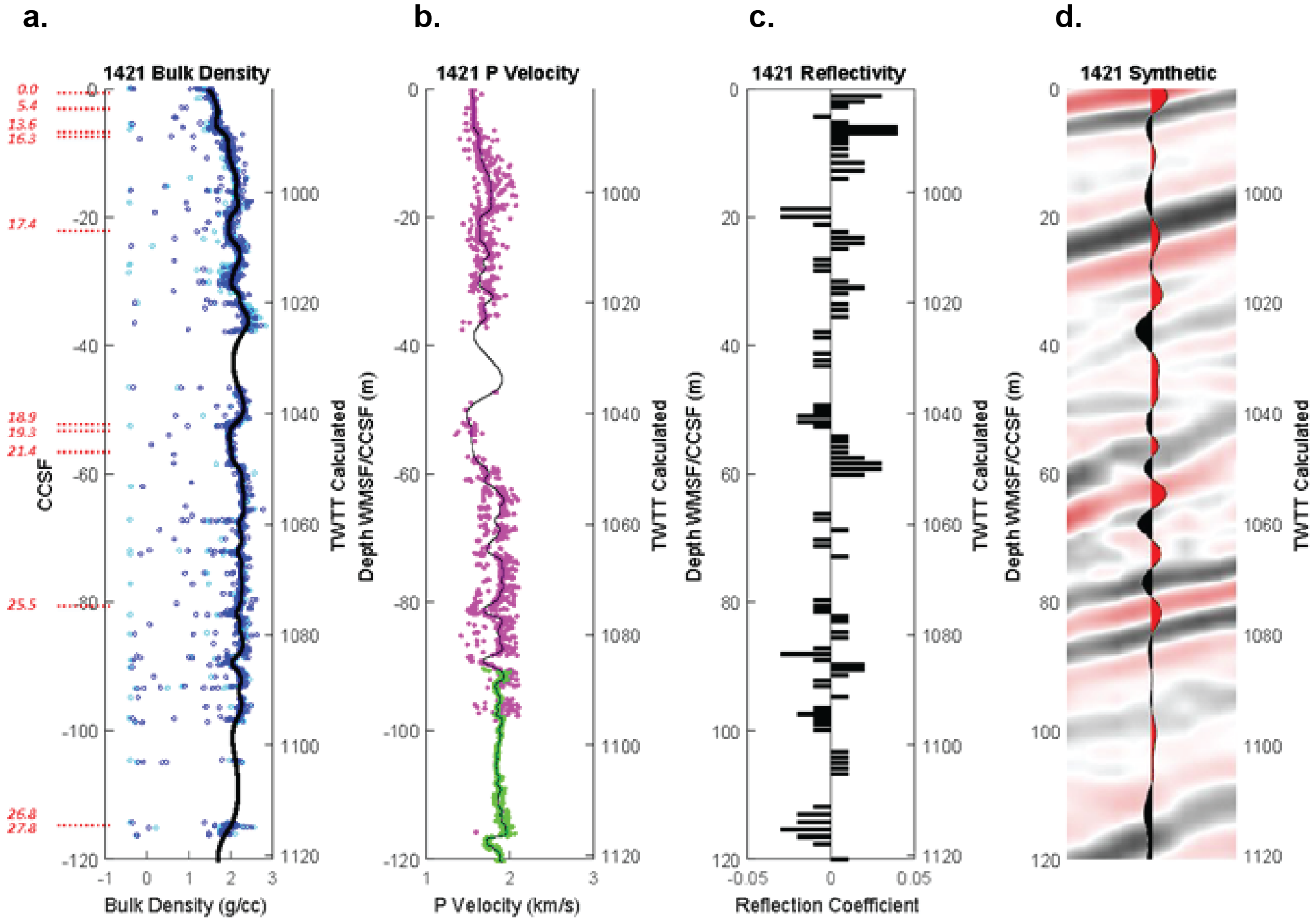

Supplementary Figure 3. Core-log-seismic integration[35] in the upper ~120 m at U1421. **a** Bulk density from whole-round (WRMSL, blue), and special task multi-sensor loggers (STMSL, cyan). Black line shows spline fit. **b** P-wave velocity from core measurements (pink, adjusted so that mean of core and log match in the zone of overlap) and from log (green). Black line shows spline fit. **c** Calculated reflection coefficient record (5 m depth bins). **d** Synthetic seismic produced from reflectivity using an extracted wavelet and resulting time-depth relationship after well tie[35] shown on background seismic data. Two-way travel time (TWTT) shown in milliseconds (ms). Red numbers to the left are ages (ka cal BP) discussed in the text.

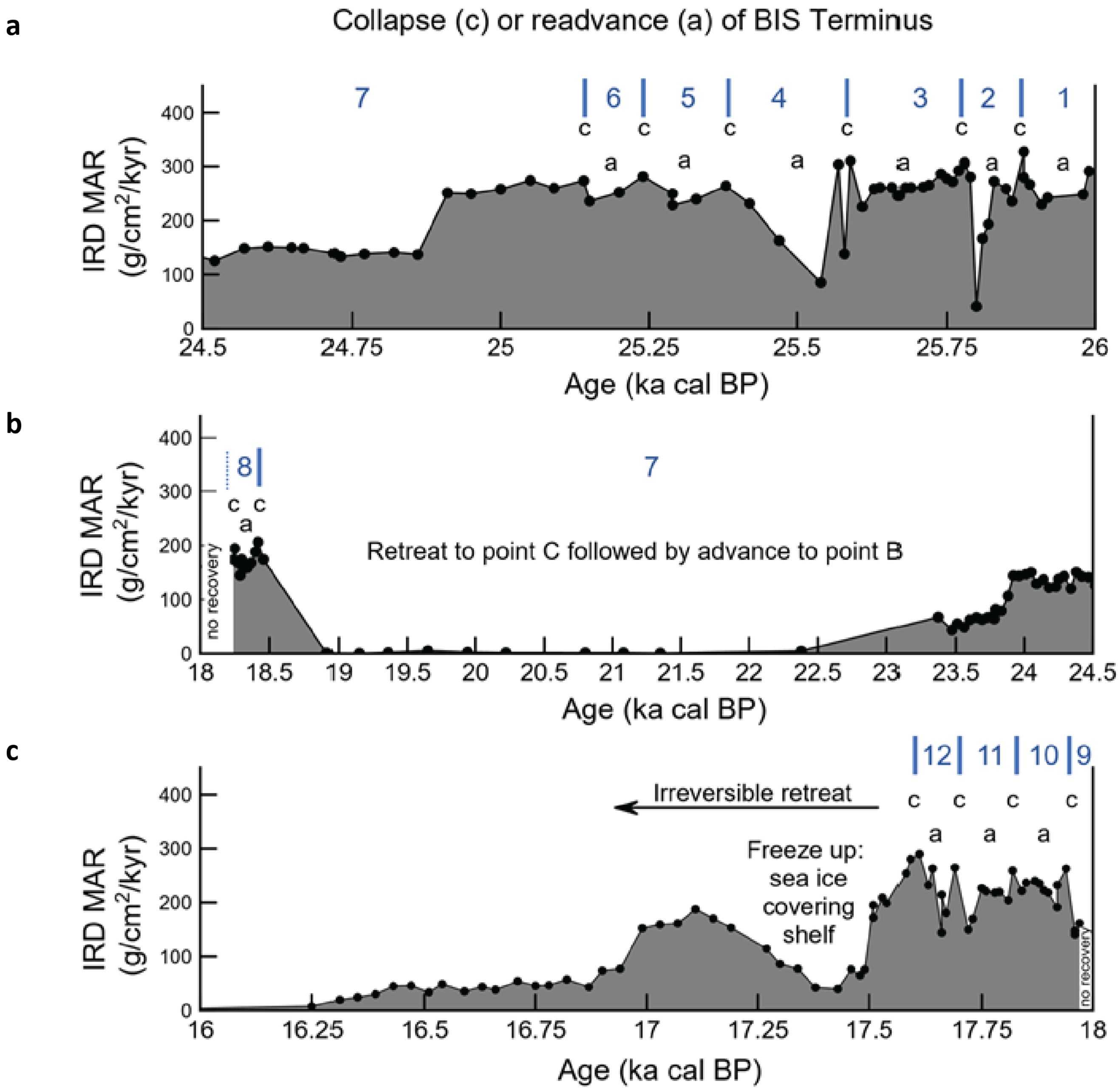

Supplementary Figure 4. Detail of selected intervals of the IRD MAR curve. **a** Early LGM: 26 to 24.5 Ka cal BP. **b** Late LGM: 24.5 to 18 ka cal BP. **c** Deglacial: 18 to 16 ka cal BP. Peaks of short duration (c) occur as icebergs are released during collapse and retreat from A to B shown on **Fig. 3b**. Readvance (a) from B to A occurs slowly as the morainal bank builds and advances, reducing calving (**Fig. 4**). Reduction in IRD MAR coupled with lower sedimentation rates is interpreted as sustained retreat from B to C (**Fig. 3b**). Final retreat begins at 17.6 ka cal BP. Retreat-advance cycles are shown by blue numbers

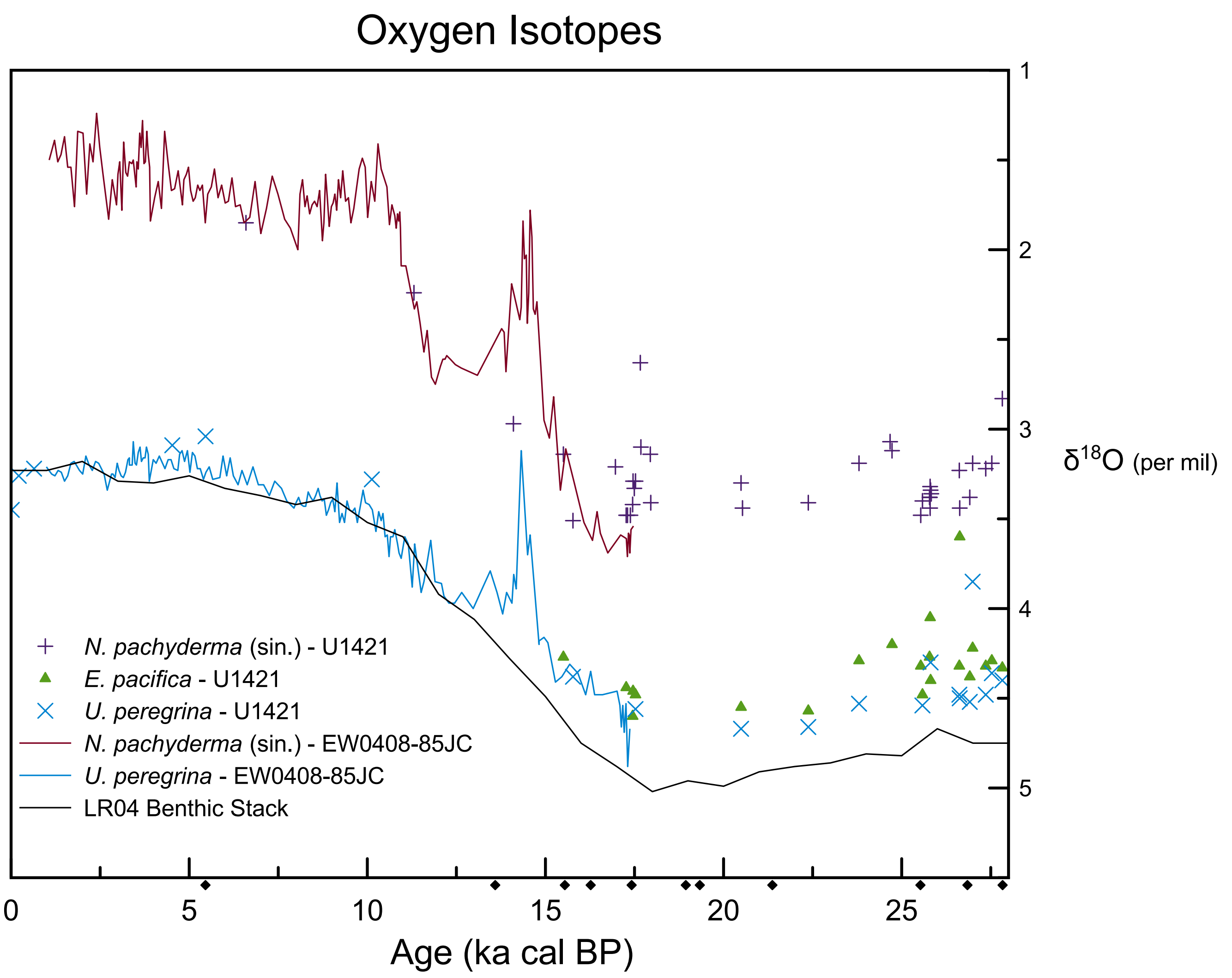

Supplementary Figure 5. Oxygen isotope analyses of planktic and benthic foraminiferal samples from U1421, along with data from site survey core EW04080-85JC[21] (**Fig. 1**) and the LR04 benthic stack[68] for comparison. Diamonds show position of radiocarbon-dated samples.

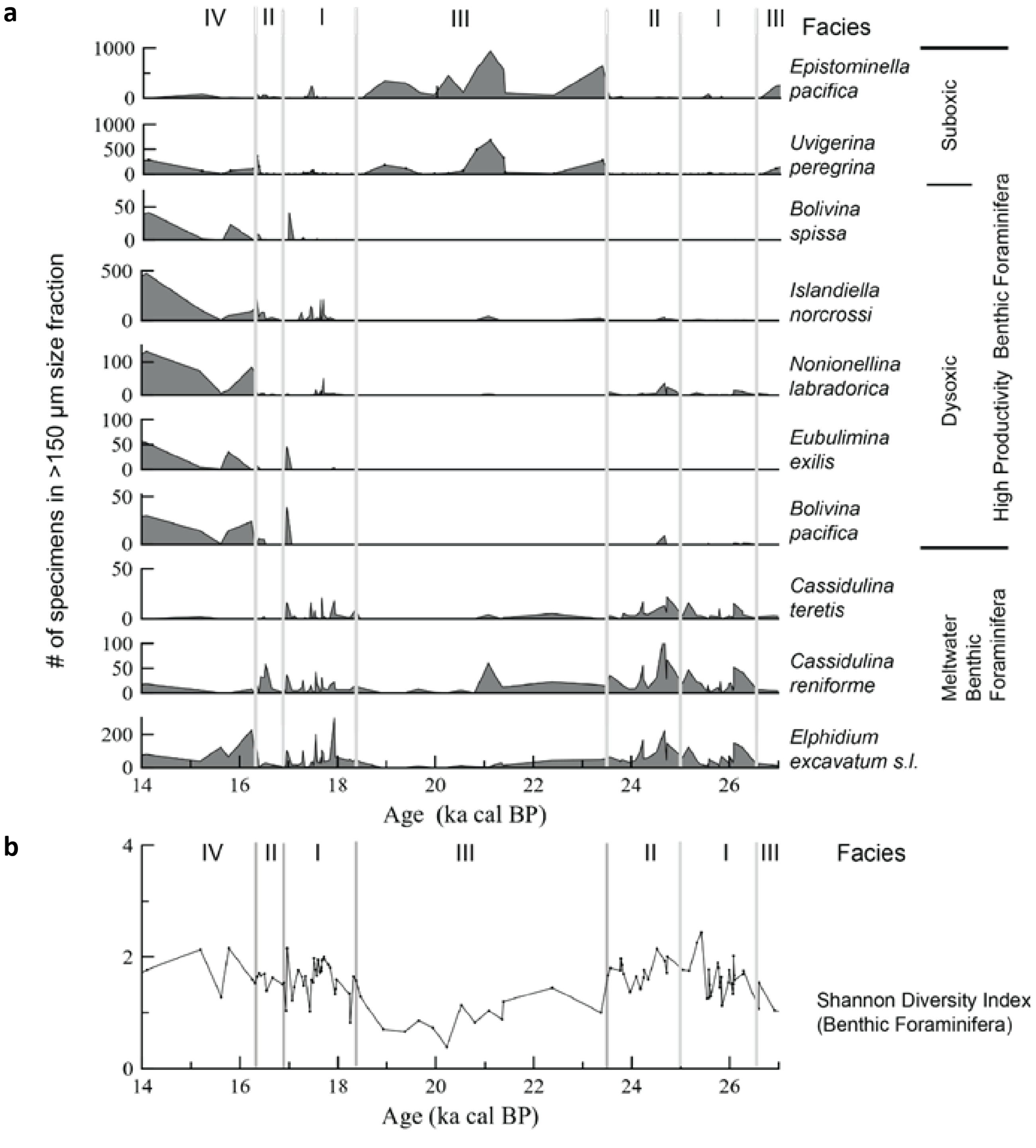

Supplementary Figure 6. **a** Distribution of most common benthic foraminifera and their paleoenvironmental significance[38-39, 69]. **b** Shannon diversity index of benthic foraminifera showing elevated diversity associated with facies I and II and lower diversity throughout facies III. Roman numerals refer to facies described in text.

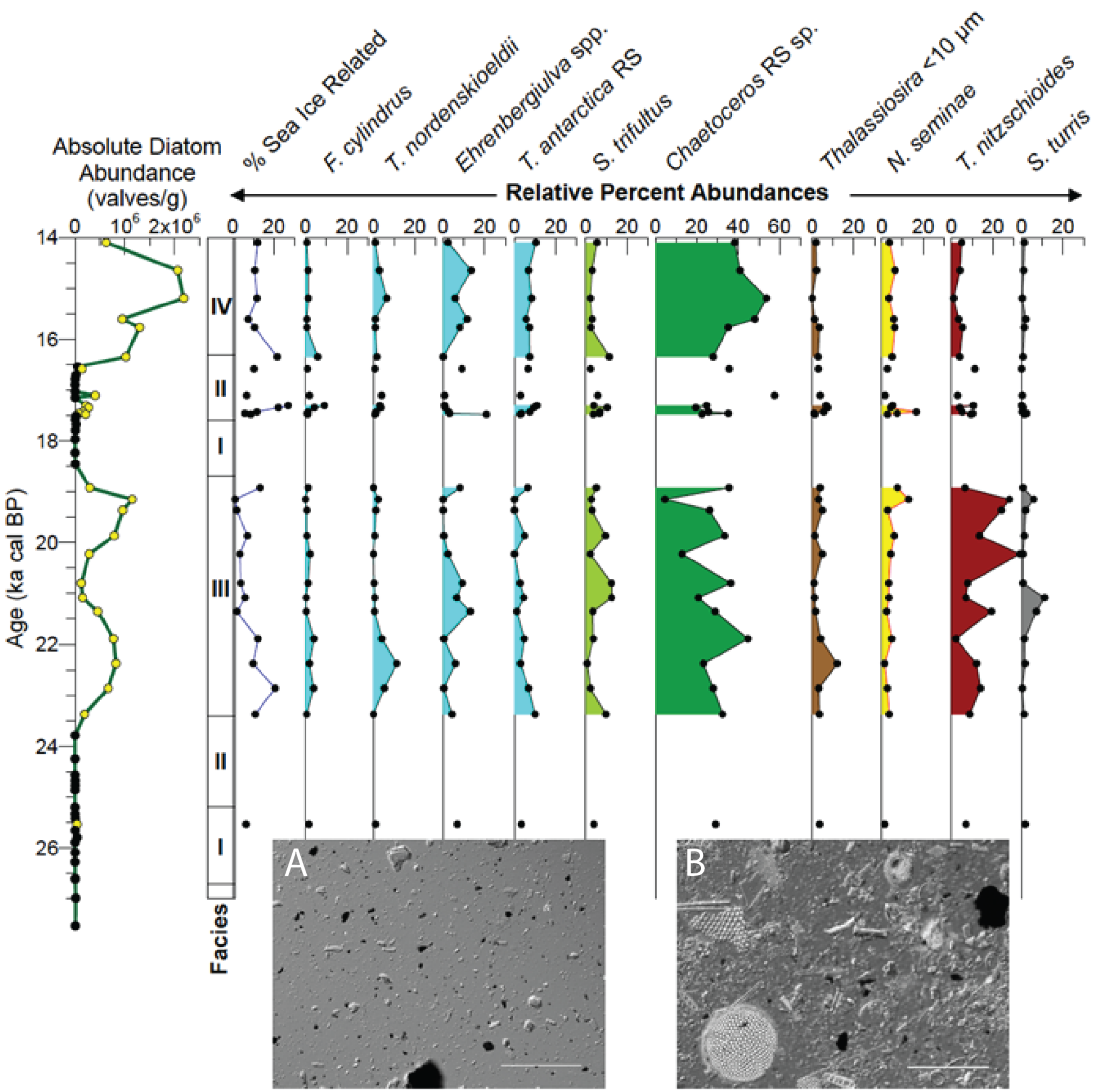

Supplementary Figure 7. Absolute diatom abundance and the most common diatoms. Black dots indicate samples with less than 25,000 diatoms per gram of sediment (considered barren, image A: U1421A 6H-3, 0-2 cm). Yellow dots indicate samples with abundant diatoms (image B: U1421C 1H-5, 88-89 cm). The % sea ice related diatoms (traditionally only *Fragilariopsis cylindrus, F. oceanica, F. reginae-jahniae, F. cylindriformis, Fossula arctica*, and *Thalassiosira antarctica* RS) were summed. Cyan shading indicates diatoms with likely sea ice affinities (*F. cylindrus, T. nordenskioeldii, Ehrenbergia* spp., *T. antarctica* var. *borealis* resting spores). Light green shading are diatoms found in regions with a dicothermal layer (*Shionodiscus trifultus*). Dark green shading indicate species associated with upwelling (*Chaetoceros* resting spores). Light brown shading indicates diatoms of unspecified affinity (small *Thalassiosira* spp. less than 10 mm in diameter). Yellow shading indicates *Neodenticula seminae*, a species associated with the open North Pacific. Red shading indicates *Thalassionema nitzschioides*, a species that is prevalent in high nutrient waters of the North Pacific, and grey shading indicates *Stephanopyxis turris*, a diatom commonly found on the shallow shelf. Scale bar in insets: 50 microns.

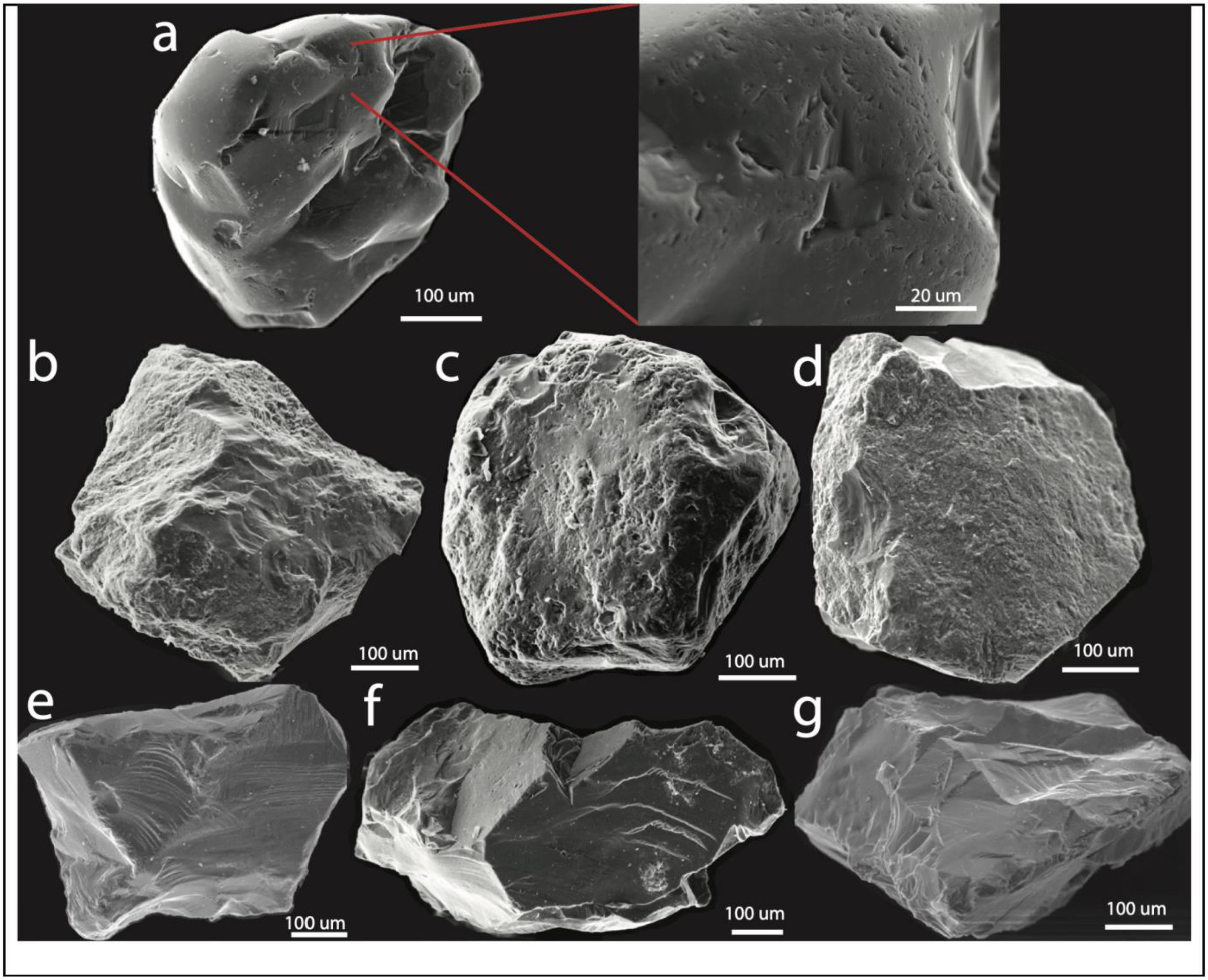

Supplementary Figure 8. SEM micrographs showing examples of **a**) grains influenced by meltwater transport including rounded edges and v-shaped percussion fractures on smooth surfaces (C-3H-2-100-102-G3), **b-d**) weathered grains classified as transported by sea ice that show smooth rounded edges, low relief with rough surfaces due to precipitation/solution (b = C-3H-4-51-53-G11, c = A-7H3-147-149-G19, d = C-3-100-102-G4), and **e-g**) glacially modified grains classified as transported by icebergs that show high relief, sharp edges, fracture faces, and arcuate steps (e = A-5H-3-146-148-G9, f = A-6H-4-51-53-G18, g = A-9H-2-0-4-G20).

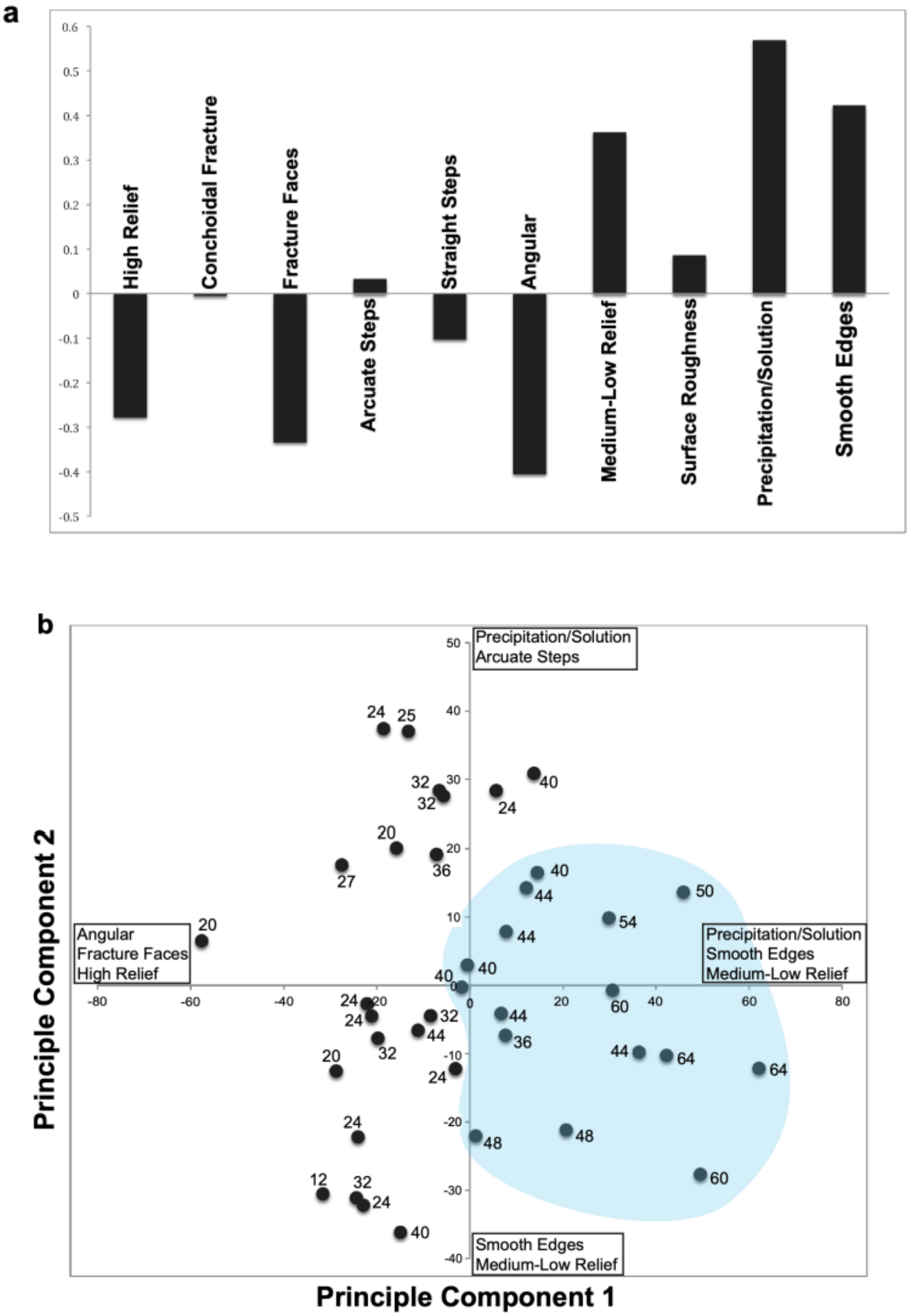

Supplementary Figure 9. **a** Loadings plot for Principle Component 1, which generally separates microtextures on quartz grains into glacially modified IRD (negative loadings) from weathered sea ice rafted debris (positive loadings). This is consistent with the literature and supports the interpretation in our southern Alaskan field area. PC l explained 38% of the variance. **b** Plot of PC l versus PC 2 for all imaged samples (explaining 61% of the variance). The percent sea ice rafted grains (determined as described in Methods) are shown next to each sample on the plot. Most samples with ≥ 40% sea-ice rafted grains plot within the shaded area.
