# Supplementary material for: Sediment controls dynamic behavior of a Cordilleran Ice Stream at the Last Glacial Maximum
Source: Nat Commun. 2020 Apr 14;11:1826. doi: 10.1038/s41467-020-15579-0 (PMC7156478; doi:10.1038/s41467-020-15579-0)
Supplement: Supplementary file 3 — Description of Additional Supplementary Files [file 41467_2020_15579_MOESM3_ESM.pdf]

Description of Additional Supplementary Files

File Name: Supplementary Data 1
Description: Ice-rafted debris

File Name: Supplementary Data 2
Description: Radiocarbon Analyses

File Name: Supplementary Data 3
Description: BChron Age Model

File Name: Supplementary Data 4
Description: Sedimentation Rates

File Name: Supplementary Data 5
Description: Stable Isotopes

File Name: Supplementary Data 6
Description: Foraminifera Checklist

File Name: Supplementary Data 7
Description: Diatom Checklist

File Name: Supplementary Data 8
Description: Geochemistry Analyses

File Name: Supplementary Data 9
Description: Quartz Microtexture Analysis
